# Innovative Long-Dose Neurorehabilitation for Balance and Mobility in Chronic Stroke: A Preliminary Case Series

**DOI:** 10.3390/brainsci10080555

**Published:** 2020-08-14

**Authors:** Catherine Boissoneault, Tyler Grimes, Dorian K. Rose, Michael F. Waters, Anna Khanna, Somnath Datta, Janis J. Daly

**Affiliations:** 1 Department of Neurology, College of Medicine, University of Florida, Gainesville, FL 32608, USA; caboissoneault@gmail.com (C.B.); anna.khanna@neurology.ufl.edu (A.K.); 2 Department of Biostatistics, College of Public Health and Health Professions, University of Florida, Gainesville, FL 32608, USA; tyler.grimes@ufl.edu (T.G.); somnath.datta@ufl.edu (S.D.); 3 Department of Physical Therapy College of Public Health and Health Professions, University of Florida, Gainesville, FL 32608, USA; dkrose@phhp.ufl.edu; 4 Brain Rehabilitation Research Center, North Florida/South Georgia VA Medical Center, Gainesville, FL 32608, USA; 5 Neurovascular Division and Stroke Program, Department of Neurology, Barrow Neurological Institute at Dignity Health St. Joseph’s Hospital and Medical Center, Phoenix, AZ 85013, USA; michael.waters@dignityhealth.org

**Keywords:** balance, coordination, function, gait, mobility, quality of life, stroke

## Abstract

(1) Objective: The objective was two-fold: (a) test a protocol of combined interventions; (b) administer this combined protocol within the framework of a six-month, intensive, long-duration program. The array of interventions was designed to target the treatment-resistant impairments underlying persistent mobility dysfunction: weakness, balance deficit, limb movement dyscoordination, and gait dyscoordination. (2) Methods: A convenience sample of eight chronic stroke survivors (>4 months post stroke) was enrolled. Treatment was 5 days/week, 1–2.5 h/day for 6 months, as follows: strengthening exercise, balance training, limb/gait coordination training, and aerobic exercise. Outcome measures: Berg Balance Scale (BBS), Fugl-Meyer Lower Limb Coordination (FM), gait speed, 6 Minute Walk Test (6MWT), Timed up and Go (TUG), Functional Independence Measure (FIM), Craig Handicap Assessment Rating Tool (CHART), and personal milestones. Pre-/post-treatment comparisons were conducted using the Permutation Test, suitable for ordinal measures and small sample size. (3) Results: For the group, there was a statistically (*p* ≤ *0*.04) significant improvement in balance, limb movement coordination (FM), gait speed, functional mobility (TUG), and functional activities (FIM). There were measurable differences (minimum detectible change: MDC) in BBS, FM, gait speed, 6MWT, and TUG. There were clinically significant milestones achieved for selected subjects according to clinical benchmarks for the BBS, 6MWT, gait speed, and TUG, as well as achievement of personal milestones of life role participation. Effect sizes (Cohen’s D) ranged from 0.5 to 1.0 (with the exception of the (6MWT)). After six months of treatment, the above array of gains were beyond that reported by other published studies of chronic stroke survivor interventions. Personal milestones included: walking to mailbox, gardening/yardwork, walking a distance to neighbors, return to driving, membership at a fitness center, vacation trip to the beach, swimming at local pool, returning to work, housework, cooking meals. (4) Conclusions: Stroke survivors with mobility dysfunction were able to participate in the long-duration, intensive program, with the intervention array targeted to address impairments underlying mobility dysfunction. There were either clinically or statistically significant improvements in an array of measures of impairment, functional mobility, and personal milestone achievements.

## 1. Introduction

Stroke is a leading cause of long-term disability worldwide, with annual frequency of stroke at 15 million, of which 5 million are permanently disabled [1]. This disability extends chronically, with 26% to 33% of stroke survivors exhibiting mobility dysfunction 1–15 years after stroke [2,3,4]. Even after standard rehabilitation has been completed, there is an unmet need in the realms of social reintegration, health-related quality of life, maintenance of activity, and self-efficacy for chronic stroke survivors, all of which are dependent upon functional capability that is not restored [5]. These problems persist due, essentially, to a gap in care. That is, there is ineffectiveness in standard care at the time of treatment such that stroke survivors do not reach their maximum potential, and/or there is absence of long-term care or rehabilitation to maintain or to improve function into the chronic, life-long phase. Supporting this point, studies have recorded important information for chronic stroke survivors, regarding those who did not receive treatment in the chronic phase and who exhibited declines in strength, balance, and mobility (e.g., [6]). Some exhibited no change in limb dyscoordination, imbalance or poor endurance and physical fitness (for example, according to measures of V02 max (e.g., [7])). Current standard care in the chronic phase is either minimal or no care.

One problem encountered in stroke research is that many studies have reported results that have targeted treatment to address one or two impairments, for example weakness (e.g., [8]), balance dysfunction (e.g., [9]), or physical endurance (e.g., [7]); though these types of studies are very important additions to the literature, there remains a gap in the literature. That is, these very focused treatments were not intended to and do not produce the gains needed in the full array of factors underlying mobility dysfunction and the resulting poor quality of life; the array of factors which underlie mobility dysfunction can include the following: limb dyscoordination, weakness, balance deficits, gait dyscoordination, and poor physical endurance [10]. Therefore, the first purpose of the current work was to develop and administer an intensive neurorehabilitation mobility/fitness program containing an array of interventions for chronic stroke survivors, entitled “Safety, Functional Outcomes, and Recovery after Stroke (SOARS)”; this SOARS program was designed to target a fuller array of impairments preventing recovery of mobility [11], and was, therefore, composed of strengthening, balance training, limb movement and gait coordination training, and aerobic exercise.

A second problem encountered in stroke research, to date, is that studies with shorter duration treatments (<6 months) do not provide data that show that a plateau has been reached in terms of “maximal potential reached” in response to the respective treatments administered. Longer-duration treatment and monitoring could address this gap in the literature. In a “catch-22” situation (no-win situation), possibly because of the cost of research studying long-duration treatments (≥6 months), there is a paucity of studies with longer duration treatments; however, the evidence from these types of studies is needed in order to support the expenditure of clinical resources in long-duration interventions for stroke survivors. There are two long-duration studies of note. First, Batchelor et al. [12] utilized a 12-month program (3–5 visits/week, 30–40 min, focusing on balance training) with weekly follow up by phone, and found no difference in balance compared to those who received usual care [12]. We can note that this study did not describe addressing the array of impairments possibly underlying the balance deficits; this gap emphasizes the importance of our study “first purpose” (i.e., provide an array of interventions to address mobility deficits). In a second important long-duration study, Stuart et al. [13] administered a 6 month walking, strength, and balance training program (3 visits/week, 1 h) and found a 5.1 point improvement in the Berg Balance Scale (BBS) which was statistically significant and exceeded minimum detectible (measurable) change (MDC), but only a 0.07 m/s change in gait speed, which is below the MDC and the minimum clinically important difference (MCID) for gait speed [13]. Though these two studies offered a long-duration intervention, at the same time, they were not designed to address the full array of impairments such as poor fitness/endurance, limb dyscoordination, or gait dyscoordination, which can underlie poor balance, limitations in functional activities, and poor quality of life, nor the intensity of intervention that could be optimal for functional recovery [11]. Therefore, the second purpose of the current work was to administer the SOARS program, within the framework of an intensive, long-duration protocol.

## 2. Methods

### 2.1. Subjects

The study design was a single cohort (no control group), intervention study. A convenience sample of individuals poststroke was enrolled in the neurorehabilitation mobility/fitness SOARS program. The protocol for the study received prior approval from the University of Florida Institutional Review Board (IRB, protocol number: RB201400568), which includes the Malcom Randall VA Human Research Protections Program (HRPP), and informed consent was obtained from each subject. Inclusion criteria included the following: ≥4 months post stroke; ability to follow two-step commands (e.g., hold the grab bar and stand on one leg); swing and stance phase gait deficits such as inability to flex the knee and ankle in the sagittal plane, in a normal manner so the foot clears the floor; inability to control normal knee angle during single limb weight bearing during the stance phase. Exclusion criteria included the following: any acute or progressive disease or diagnosis; neurological diagnosis, except stroke; lower motor neuron damage or radiculopathy; more than one stroke; Fugl-Meyer lower limb motor sub-score greater than 32; severe obesity (body mass index >35); current participation in physical therapy for gait training or cardiopulmonary rehabilitation.

### 2.2. Intervention

Soars Program components. The SOARS program was constructed of several components, each of which had been separately supported with prior study and publications. However, in prior work, each component was studied separately as a single type of treatment. The components of treatment for the SOARS were strengthening, limb coordination training, balance training, gait coordination training, and fitness training.

Strengthening exercises were administered for lower limb muscles at up to 50–80% of the 1 repetition maximum (1RM), up to 6–12 reps per set, and up to 1–4 sets per muscle group [14,15,16]. The methods for balance training were conducted according to standard physical therapy practice [16,17]; exercises included two-legged standing weight shifts across the plantar surface of the feet; one-legged standing weight shifts across the plantar surface of the foot; two-legged and one-legged balance practice on a variety of challenging surfaces. Yang-style Tai Chi movements [18] were integrated into the program, using standard clinical practice principles for treatment progression [10,19,20]. The Tai Chi “moves” served to retrain slow weight shift from one limb to another in a variety of directions, adding the balance challenge of slow arm movements. Standard balance training subsequently employed greater balance challenges such as larger torso movements during one-leg standing and weight shifts and imposed balance disturbances for practicing faster-speed balance recovery strategies [14,15,16].

Moderate intensity aerobic training was conducted based on the published protocol and methods of Quaney et al. [7]. The exercise program was on a stationary bicycle, with the expectation that subjects would eventually achieve cycling twice weekly, up to a target of 70% of maximal heart rate for 35 min, with a warm up (5 min) and cool down (5 min). Subjects began the first week with less than 10 min of cycling (and with a cap of only 40% of projected target heart rate, which was not reached by most subjects in the first weeks); in fact, some subjects were able to cycle only 3 min on a given day during their first weeks, before they reported fatigue and the session was concluded for that day. As soon as participants were first able to reach that initial cap of 40% heart rate reserve [21], they underwent graded exercise testing with electrocardiographic (ECG) monitoring, administered by an experienced cardiovascular researcher certified in exercise stress testing and under the supervision of a cardiologist, who determined each given subject’s safety in being further progressed within the planned aerobics cycling exercise (guidelines for stroke survivors; [21,22]). Subjects were progressed by only several minutes across sessions. Heart rate, blood pressure, perceived exertion (RPE), and potential side effects were closely monitored during each training session to ensure safety. Cycle exercise was not performed on a given day if resting blood pressure was greater than 140/90.

The methods for coordination training and gait coordination training and neurorehabilitation treatment progression were conducted according to prior published work [10,19,20], and based on the phenomenon of brain plasticity and motor learning principles, including the following: motor task-specific practice (practice as close-to-normal movement as possible [23,24,25,26] with continual progression toward normal); high repetition of the desired movement pattern [27,28,29]; focused attention [30], training specificity [28,31,32,33], and awareness and feedback [34].

**Figure 1:**
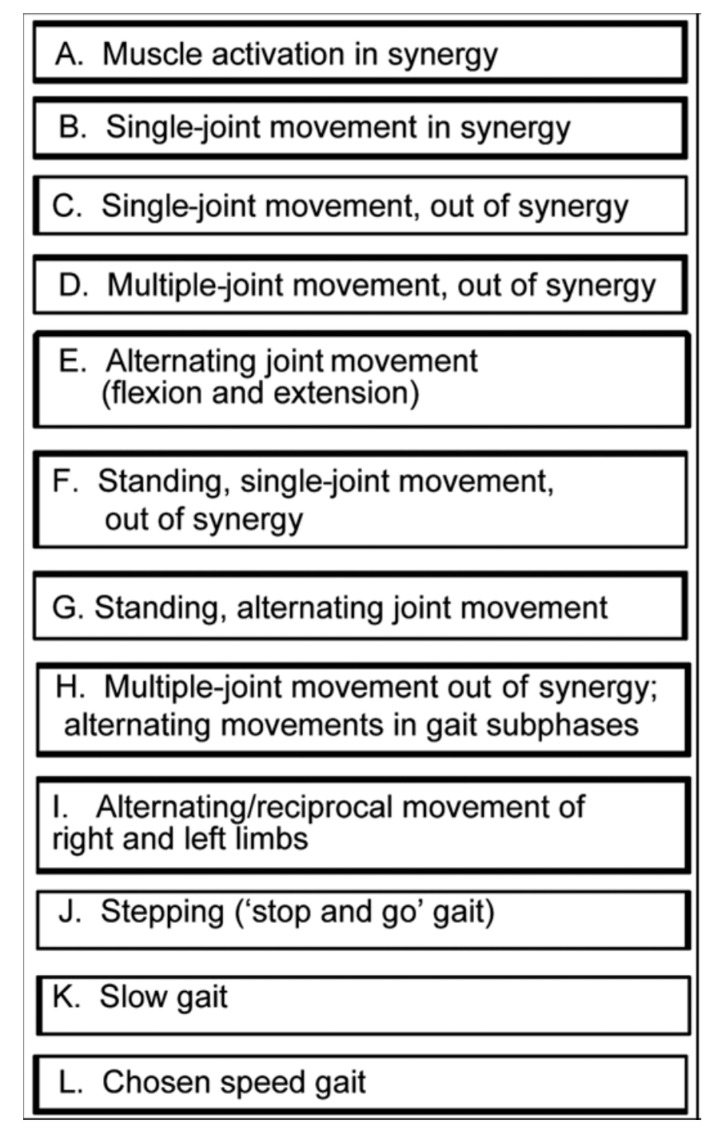
Motor Task Hierarchy for Limb and Gait Coordination Training. Key: In Synergy: with all the given limb joints flexed or all the limb joints extended. Out of Synergy: with at least one joint flexed while all other joints in the limb are extended. Stop and Go Gait: Practice of one subphase of the gait cycle several times in place, before moving forward through that subphase of the gait cycle. Gait subphase: Swing and Stance Phases divided into subphases such as heel-off to toe-off.

At the first treatment sessions, an assessment was made to identify the set of baseline exercises that would be initially assigned. To determine the difficulty of initial exercises, we considered muscle strength, coordination, muscle tone, balance, gait coordination, gait speed, and function. Critical details included the following: ability to generate normal movement at the hip, knee, and ankle for the types of motor tasks listed in Figure 1; for those movements that were impaired, assessment included not only the ability to volitionally generate each of these movements, but also the ability to move in conjunction with assist devices (functional electrical stimulation (FES), parallel bars, walker, cane) Each motor task in Figure 1 was assessed in more detail for the following characteristics:

- Percentage of the normal range of movement that could be executed, volitionally and independently.
- Percentage of the motor task that could be executed with the support of verbal or tactile facilitation.
- Percentage of the normal range of movement that could be executed along with an assistive movement device.
- Normality of effort level during the task (e.g., holding breath, abnormal co-contraction of muscles distant from the targeted task joints or antagonist muscle contractions).
- Compensatory strategies employed during execution of the motor task.
- Percentage of the task for which compensatory strategies were employed.
- Number of repetitions of the motor task that could be performed with only a “beat” between repetitions before the motor task was performed in an abnormal fashion. ([20], page xix).

For motor task practice, body position is important in coordination relearning; the initial position is selected according to coordination capability and the functional goal. Awareness training was administered so that the stroke survivor could learn to identify and differentiate between normal coordinated movement and the abnormal movement [34]. When volitional movement is abnormal, repeating it during practice is counterproductive. In that case, for ankle and knee motor practice, we employed surface FES to assist in producing a more coordinated movement. FES-assisted movement enables the practice of a movement task earlier in the rehabilitation process than would be possible otherwise through only volitional effort [10,35,36]. The surface FES device we used was an EMS+2^™^ Staodyn, Inc (Longmont, CO, USA). For the FES-assisted exercise, the parameters were as follows: amplitude at subjectively comfortable level; 30 Hz; 300 ms pulse width; duty cycle, 5 s on and 5 s off. For gait training, the duty cycle was adjusted to the timing of the individual gait cycle or a foot switch was used. FES was employed for the practice of ankle dorsiflexion and eversion and knee flexion. Speed of movement practice was progressed as capability for performing a given movement pattern in a coordinated manner improved. Treatment was progressed according to following rules:

1. 50 percent of normal range of movement is executed, volitionally, independently; or 50 percent of motor task is executed with support of verbal or tactile facilitation; or 50 percent of normal range of movement is executed, along with a motor assist device.
2. Normal level of effort is expended during task (no holding breath or associative reactions in other limbs or trunk; relaxed uninvolved muscles).
3. If motor compensatory strategies are employed, less than 10 degrees of movement is performed that is compensatory in nature.
4. If motor compensatory strategies are employed, at least half of motor task is performed without compensatory strategies.
5. Five or more repetitions of motor task can be performed in a row with only a “beat” between before motor task deteriorates into uncoordinated or incorrect. ([10], page xxi)

Greater detail and examples are provided in the Supplementary Materials on Limb and Gait Coordination Training, in the section of the document entitled, “Implementation”.

Schedule: The SOARS program was administered for six months by a physical therapist in an outpatient setting, 5 days/week, 1–2.5 h/day. The schedule for the components was as follows: strength training 3 days/week; limb and gait coordination training daily; balance training 2–3 days/week; aerobic training on a recumbent cycle 2 days/week.

### 2.3. Measures

Assessments were conducted by the trained study therapist, at baseline and at the end of 6 months treatment. Impairment measures included: the Berg Balance Scale (BBS), which exhibits a minimal detectible change (MDC) of 4.13 points [37] and a threshold for functional independence of 45 points [38]; the Fugl-Meyer-Lower Extremity scale (FM-LE) which has an MDC of 3.57 points [39]. Functional mobility measures were as follows: gait speed, calculated as the walking speed during the second minute of the 6MWT (gait speed MCID = 0.16 m/s [40,41]; 6 Minute Walk Test (6MWT), minimum clinically important difference (MCID), 113 ft; Timed Up and Go (TUG; (MDC, 4.13 s [39,42]; threshold for fall risk in older stroke survivors, 14 s [43]. Functional activities were assessed using the overall Functional Independence Measure (FIM; MCID, 22 points [44]), a self-report measure. Quality of Life (or life role participation) was assessed using the Craig Handicap Assessment and Reporting Technique (CHART; total score norm for chronic stroke, 425.8 points [45].

### 2.4. Data Analysis

Descriptive statistics were generated (mean, range, standard deviation) to inspect whether there was achievement of pre-/post-treatment change equal or greater than MCID or MCD and achievement of functional thresholds. Pre-/post-treatment comparisons were conducted using the Permutation Test [46], which is suitable for a small sample size, non-normal distributions, and ordinal measures. The effect size of the change was computed using Cohen’s d [47]. Confidence intervals were computed using the percentile bootstrap method [48]. Finally, raw data were plotted for each participant for each measure and are presented in the figures below.

## 3. Results

Subjects were in the age range 46–80 years. They were 50% male/female. All subjects were ≥7 months poststroke, with the exception of subject number 3 (4 months). Other participant characteristics are provided in Table 1.

### 3.1. Impairment

**Figure 2:**
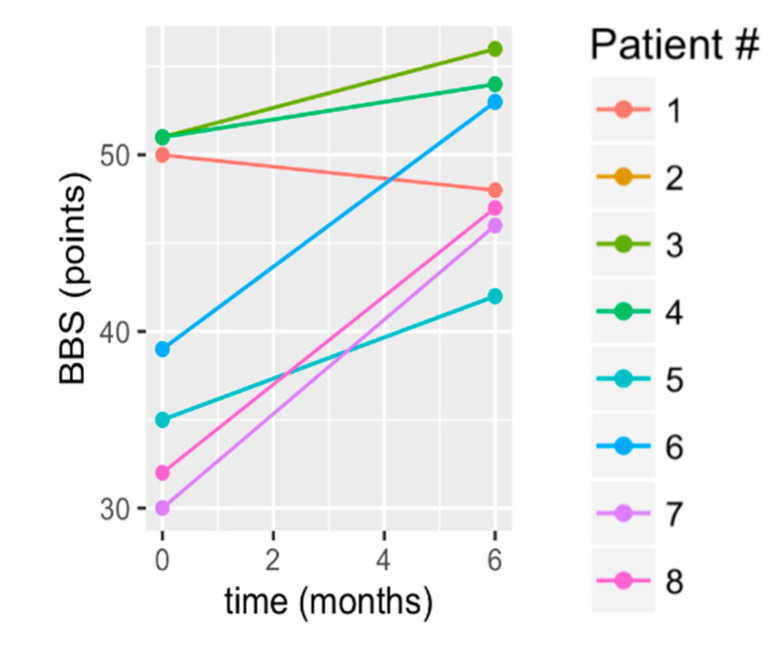
Change in Berg Balance Scale (BBS) from baseline to 6 months. S2 and S4 had identical baseline and 6 month scores, which is why S2’s data are obscured beneath S4’s data.

There was a statistically significant improvement in balance (BBS mean change: 7.88 +/− 6.05 points; *p* = 0.016; Table 2). Since there was a small sample size, we should note the following from The raw data in Figure 2 showed that one subject had a decline (S1); three subjects had the greatest gains (S6–S8); three subjects had gains that were slightly bit less striking (S2–S5). Additionally, by 6 months of treatment, 75% of participants exceeded the BBS MDC; Table 3, Figure 2). Most notably, from a clinical standpoint, of the four participants whose baseline BBS scores were <45/56, 75% exceeded the BBS threshold for functional independence at post treatment (Table 3).

**Figure 3:**
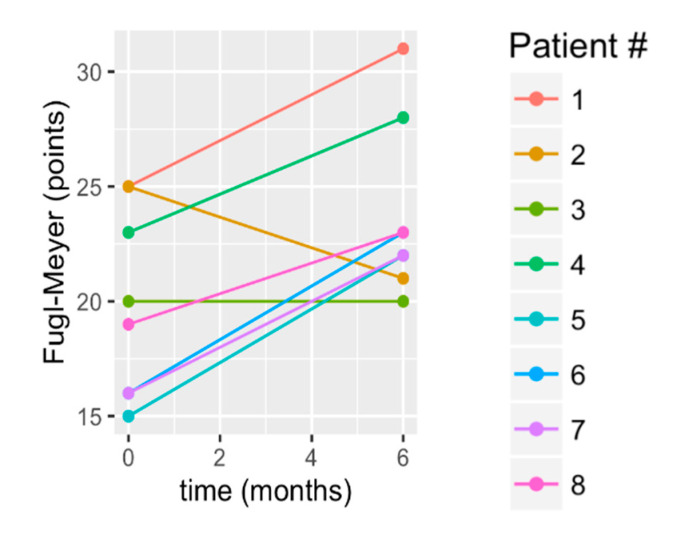
Change in lower extremity Fugl-Meyer score from baseline to 6 months. Key: Fugl-Meyer; (LE): Lower Extremity

Lower extremity coordination improved significantly (FM mean change: 3.88 +/− 3.66 points; *p* = 0.047) with 75% of participants exceeding the MDC (Table 3, Figure 3). The raw data from Figure 3 show that S2 had a decline and S3 essentially maintained FM score. The remaining six subjects exhibited a similar amount of gain, observable from the similar slope of the data from pretreatment to 6 months, for those subjects in Figure 3.

### 3.2. Functional Mobility

**Figure 4:**
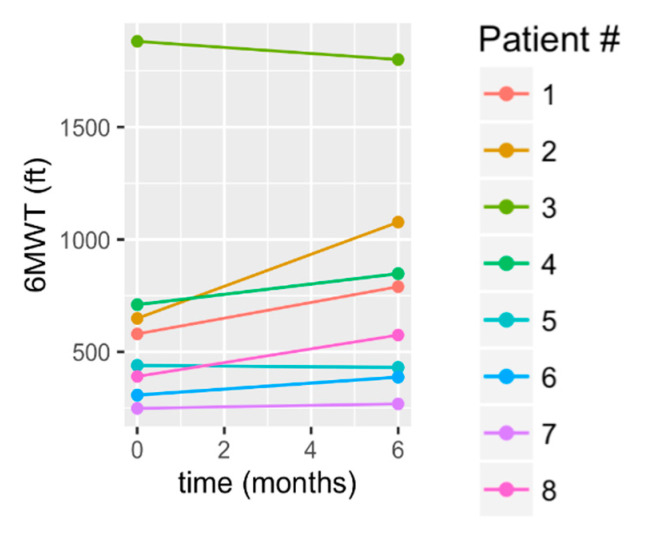
Change in 6 min walk test (6MWT) from baseline to 6 months.

The 6MWT demonstrated group maintenance at 6 months (mean change: 121 +/− 148 ft, *p* = 0.063). The raw data in Figure 4 show that S1, S2, S4, and S8 (50% of subjects) showed clinically significant gains, in that they exceeded the MCID threshold (Table 3). At the same time, there was a decline shown in S3 and S5, a slight gain in S6, and, essentially, maintenance by S7. Of those who exceeded the MDC and MCID, the mean improvement was 240 +/− 129 ft.

**Figure 5:**
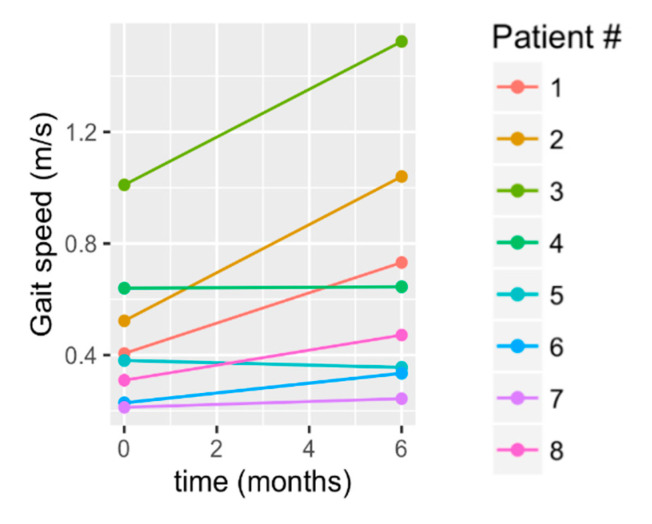
Change in gait speed from baseline to 6 months.

There was a statistically significant improvement in gait speed (mean change: 0.21 +/− 0.21 m/s; *p* = 0.023; Table 2; Figure 5). The pattern of improvement is shown in the raw data of Figure 5, in which S1, S2, S3, and S8 showed the greatest improvement. Notably, from a clinical standpoint, these four subjects, who compose 50% of subjects, exceeded the minimum clinically important threshold (MCID; Table 3; Figure 5). Additionally, notable from a clinical standpoint, 25% transitioned to higher ambulatory categories, according to Perry’s velocity-based community ambulation classification [41]. That is, one individual transitioned from a household ambulator (<0.4 m/s) to a limited community ambulator (0.4–0.8 m/s), and one improved from a limited community ambulator to a community ambulator (>0.8 m/s).

**Figure 6:**
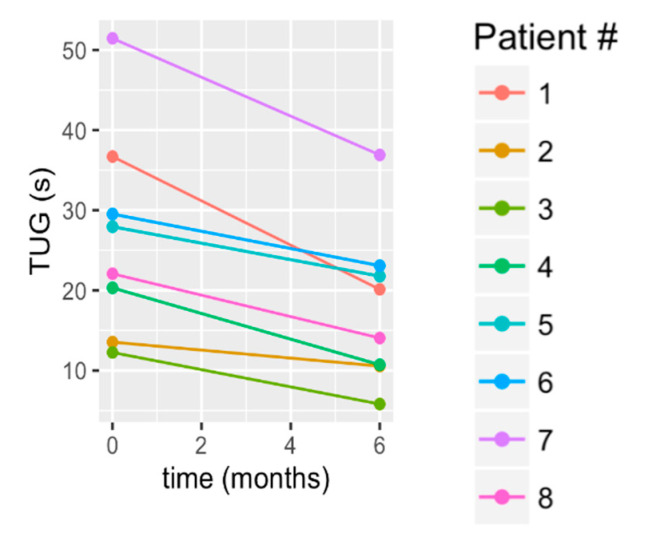
Change in the Timed Up and Go (TUG) test from baseline to 6 months.

The TUG functional mobility, showed a statistically significant improvement (mean change: 8.85 +/− 4.28 s; *p* = 0.008), with 88% of participants exceeding the MDC by an average of 9.69 +/− 3.91 s (Table 3). The raw data in Figure 6 show that S1 and S7 had the greatest improvement, followed by S4 and S8, and then S3, S5, S6 showed lesser gains, with S2 maintaining. Notably from a clinical standpoint, three subjects improved their fall risk (Table 3) by achieving the TUG benchmark for a lesser fall risk.

### 3.3. Functional Activities

**Figure 7:**
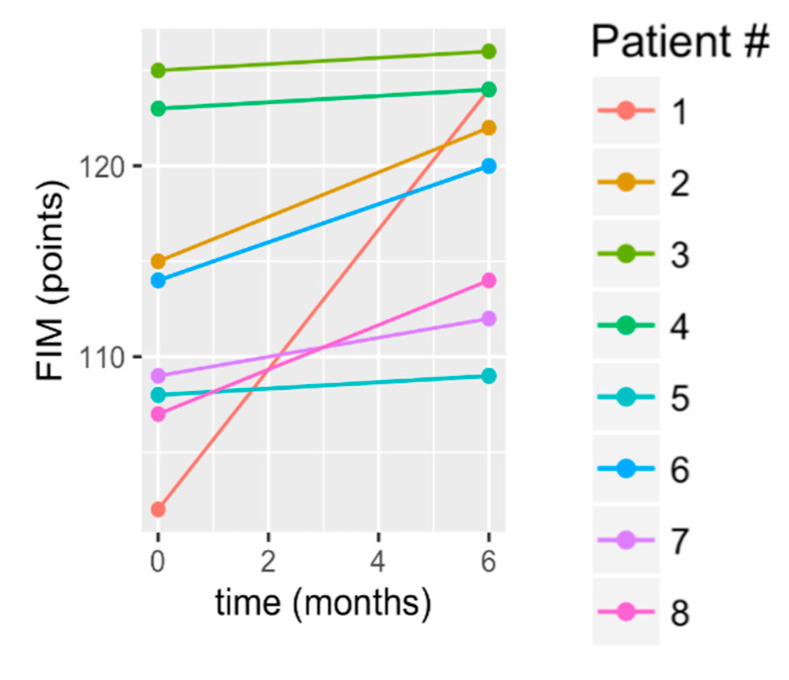
Change in the Functional Independence Measure (FIM) from baseline to 6 months.

According to statistical testing, function significantly improved, according to the FIM score, by an average of 5.86 +/− 6.54 points (*p* = 0.008; Table 2). The individual scores in Figure 7 show that S1 had greater gains than all other subjects. Others showing gains were S2, S6, S8, and S7. The remaining three, S3, S4, and S5, essentially maintained. Because of the greater gain for S1, we provide additional information for S1. We can note that, despite his disability, S1 arrived by airplane, alone, to be screened and subsequently to participate in the study. He arranged an apartment for himself for the six-month study and took a shuttle or a bus to the clinic where the study was conducted. He assumed responsibility for his own meals and self-care. Given his initial limb dyscoordination (FM score), slow gait speed, and baseline TUG scores, all of this would have been quite difficult for him and would have accentuated his functional disabilities, as evidenced by his self-reported FIM score at baseline (FIM score was 102 at baseline and improved to 124 by month 6). His baseline FIM was the lowest of the group and his 6 month score improved to less than or equal to two other subjects. This improvement could have occurred due to a number of factors. His situation forced him to walk to the grocery store and to study appointments, and he reported exercising on his own at home in the evening and on weekends. It is reasonable to consider that his improvement in limb coordination (FM), gait speed, and functional mobility (TUG) may have been the underlying factors for the improved FIM score.

### 3.4. Quality of Life

**Figure 8:**
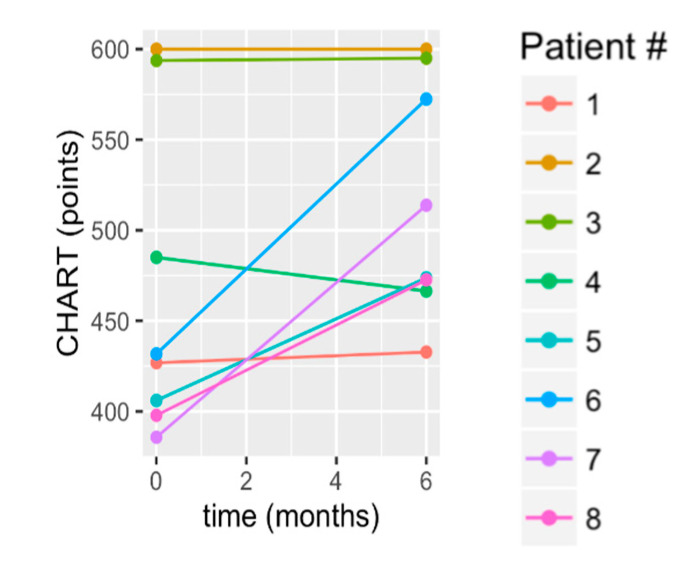
Change in Craig Handicap Assessment and Reporting Technique (CHART) from baseline to 6 months.

All participants exceeded the published CHART total norm for chronic stroke survivors at 6 months (CHART stroke norm = 425.83; Table 3), with an average improvement of 50.0 +/− 61.9 points. Since the *p*-value = 0.078, we can say that as a group, they maintained. The medium effect size, though, is understood through the individual scores shown in Figure 8 for which there were gains for 50% of the subjects (S5–S8). There was maintenance by S1–S3, and decline in S4. Notably, participants reported achieving a number of life role milestones that were important to them and not possible before participation in the study. These included walking to the mailbox, gardening/yardwork, walking a distance to neighbors, return to driving, membership at a fitness center (pool exercise and land workout), vacation trip to the beach, swimming at local pool, returning to work, housework, and cooking meals. For the most part, participants began aerobic exercise with minimal ability (3 min, for example, on the bicycle). As a result of the long-term incremental progression of cycling exercise, their endurance and aerobic safety indicators improved. Subsequently, four participants (50%) began aerobic and strength training at a local gym as a result of their improved exercise endurance, confidence in their mobility, and new experienced awareness of the benefits of aerobic and resistance exercise.

### 3.5. Adverse Events, Attendance, and CoMorbidities

There was one instance of knee pain reportedly associated with an old injury, which was resolved with medical care. There were no other study-related adverse events. Other events, not related to the study included one subject’s diagnosis of breast cancer, surgery, and radiation treatments, which interfered with attendance across some weeks (S7). A second subject (S5) experienced depression during the study but was able to respond to combined medical care and the study exercise protocol, which together, mitigated the depression. The group average attendance was 3.3 visits/week, with 75% of subjects attending at least an average of 3 visits per week across six months (Table 4). If we frame this within our own experience of working out, three times per week is a good performance in terms of showing up and working out for 1–2.5 h every week for six months. Notably, three subjects attended an average of 3–4 times weekly (first three rows of Table 4).

Subjects encountered the following expected situations which were resolved during the course of the study: difficult (e.g., bus) or unreliable transportation; work responsibilities (two participants worked); unsupportive spouse; family/childcare responsibilities. Over a six-month period, stroke survivors can experience multiple health problems. Participation in this intensive neurorehabilitation program afforded the opportunity for the therapist to maintain close evaluation of emerging problems, with referral to any needed outside specialists. During the course of this study, emerging problems were addressed, curtailed, and in some ways, supported by the SOARS program and strongly supported through referral to other professions. Examples include the following: inappropriate nutrition was corrected in three participants, with resulting improved participation in study exercises and physical endurance; inactivity from depression was improved; inactivity from mobility dysfunction was improved, with resulting mitigation of joint pain and improved range of movement; cancer-related fatigue was managed with carefully titrated aerobic activity; elevated blood pressure was identified and addressed with referral for medication management as well as management with aerobic exercise within the SOARS program. Given the difficulties encountered by stroke survivors, it may be important to offer the opportunity for interventions 5 days/week but expect attendance to be in the range of 2–4 days/week. Creative scheduling and time-management by the therapist would be required to satisfy business “productivity” measures while adjusting for the obstacles encountered by stroke survivors over long-duration care programs. One option might be a small group or dyad (two patients) treatment paradigms.

## 4. Discussion

This study contributes to the literature in a number of ways. First, this sample of chronic stroke survivors with mobility dysfunction could participate in a long-duration, intensive intervention. Second, though this was a small sample size, clinically and statistically significant gains were realized in measures of impairment. Third, and similarly, statistically significant gains were exhibited in functional activities. Fourth, clinically important personal milestones were reached and reported in quality of life, and though this was a preliminary study with small sample size and a variety of subject responses, the quality of life measure was maintained and trending toward statistically significant improvement.

### 4.1. Other Studies of Short Duration Treatment—Single Intervention

Most chronic stroke intervention studies focus in isolation on either one or two impairments as follows: strength training (e.g., [8,49]); balance training (e.g., [9,50]); stationary cycling (e.g., [7,21]) or specific types of gait training or traditional gait training (e.g., [51,52]), over a relatively short time period (<6 months). These narrowly focused interventions are not effective in improving the impairment or dysfunction beyond the narrow impairment or dysfunction treated. For example, in a meta-analysis of gait training studies [51], there was no significant effect of gait training on functional self-care measures for those in the chronic phase. Additionally, the change scores in some of these other studies are modest or fail to meet MDCs or MCIDs for the respective measures. This is an important point because the definition of the MDC is the minimum amount of change, for a given measurement tool, required to distinguish a true performance change versus a change due to variability in performance or measurement error. Therefore, the reported mean change scores for some studies were not even a measurable performance change. In the current study, there are five measures for which the MDC is known. For those five measures, the gain score exceeded the MDC, indicating a measurable performance change. Those measurable performance changes were in balance (BBS), joint movement coordination (FM), gait speed, walking endurance (6MWT), and mobility (TUG). This result was potentially due to either or both the long-duration treatment and the combined interventions administered in the current study.

### 4.2. Other Studies of Short Duration Treatment—Combined Interventions

Some studies reported combined treatments. For example, Vahlberg et al. [53] employed a short duration intervention with a combination treatment of progressive resistance and balance training [53]. They utilized a 12 week, twice weekly group circuit training paradigm and reported change scores in BBS (4.1), gait speed (0.10 m/s), and 6MWT (110 ft). The BBS reached the MDC of 4.13. However, neither gait speed nor 6MWT reached the MDC or the MCID. In contrast, in the current study, albeit with a small sample size, the BBS change score was double the MCD (7.88 points), and there was achievement of an MCID for both 6MWT (121 ft) and gait speed (0.21 m/s) for both 6MWT and gait speed, with 50% of subjects achieving results that were higher than the MCID in both measures, respectively. We should emphasize here that the MCID is important because it is an indicator of a clinically important change. In the example Vahlberg study [53], there was no change in other measures such as quality of life. Taken together, results of such shorter-duration treatments (<6 months) and the current results suggest that a longer-duration and more intensive intervention, such as provided in the current study, could potentially produce continued and greater improvement. Confirmation of this would require further study in a future larger study design.

### 4.3. Other Studies of Long Duration Treatment (≥6 Months of Rehabilitation Intervention)—Chronic Stroke

Studies of long duration (≥6 months) are scarce and have produced mixed results. Batchelor et al. (2012) utilized a 12 month balance-focused home exercise program (3–5×/week, 30–40 min) with a weekly follow up by phone and found no difference in balance compared to those who received usual care (which in the chronic phase, can likely be no rehabilitation) [12]. Stuart et al. (2009) administered a 6 month walking, strength, and balance training program (3×/week, 1 h) and found a 5.1 point improvement in the BBS (statistically significant and exceeded MDC), but only a 0.07 m/s improvement in gait speed, which is below the MDC and the MCID [13]. In contrast, the current study, albeit with a small sample size, produced both a 7.8 point gain in BBS and a clinically significant gain in gait speed with 50% of subjects above the MCID. There are at least two differences in the currently reported protocol versus that of the prior published protocols. First, in the Batchelor protocol [12], there was emphasis on balance training, with no treatment described for endurance/fitness, which might explain the lack of change in their speed measure. Additionally, they found no difference in balance for the experimental versus the control group for the balance measure; possible explanations could be a treatment that was too short each day or a treatment that did not address the underlying impairments that precluded balance improvement, such as dyscoordination of limb movements. In the current study, there was intervention for limb movement dyscoordination, which in part, could explain the greater improvement in balance. In the Stuart protocol [13], though there was a balance gain equal to the MCID of the BBS, there was essentially no change in gait speed (below the MDC). In the current study, there was a cycling intervention for endurance/fitness, which might explain the measurable and clinically significant gains in the speed measures in the currently reported study. Taken together, results of these and the current study suggest that the broader array of intervention components could have a higher impact on balance and gait speed.

### 4.4. Function and Life Role Participation

Strength, coordination, balance, and physical endurance/fitness underlie the ability to complete functional tasks and participate in life role activities. Others have not reported on measures of function or have reported no significant gains in functional task performance for chronic stroke survivors (e.g., [12,51]). In the current protocol, we provided a carefully administered exercise progression for balance, coordination, strength, and endurance/fitness training; we note, again, the caution of our small sample size, before stating that 75% of subjects exhibited measurable improvement (>MDC) in both balance (BBS) and limb coordination (FM-LE). The improved lower limb coordination and balance may have contributed to some gains in gait speed ([54]; 50% of subjects exceeded the MDIC in gait speed), which could, in turn, support the achievements of personal milestones in function and quality of life. Schmid et al. [55] found that a gain in gait speed which facilitates transition into a higher ambulatory category can lead to significant functional and quality of life improvements ([55] Schmid).

### 4.5. Standard Care and Decline of Mobility and Function

To appreciate the significance of these improvements, it is helpful to consider the current state of chronic stroke research and clinical care. Chronic stroke survivors receiving “usual care” (in most cases, yearly follow-up with a neurologist or primary care provider) do not improve with time [6,7,9]. For example, Stuart et al. (2009) followed 48 chronic stroke survivors over 6 months and found a −0.047 +/− 0.019 m/s decrease in gait speed and a −1.5 +/− 1.7 point decrease in BBS [6]. Quaney et al. (2009) followed 19 chronic stroke survivors over 8 weeks who completed stretching exercises thrice weekly and found the following information: a decline in TUG scores of −4.01 s; a difference in FM (total) score of only 2 points (<MDC); a decline in Vo2Max of −0.28 mL/(kg min) [7]. Recent reviews and meta-analyses of intervention studies suggest that functional recovery in chronic stroke populations is possible but requires sufficient treatment intensity and duration [56,57] that is not currently available in standard care.

In this current preliminary work, the SOARS program results could be understood from a number of perspectives. The SOARS program is a comprehensive mobility/fitness neurorehabilitation program composed of the components necessary to improve mobility and function, with treatment components selected to address chronic stroke impairments underlying mobility dysfunction. For this, we employed treatment components, each separately somewhat supported by previous studies. However, there are three differences in the SOARS, as follows: (1) an array of combined, integrated interventions; (2) administered at a greater frequency; (3) longer duration than previously studied. The treatment components of strengthening, balance training, gait coordination training, and aerobics/fitness training were selected for their complementary advantages and for their breadth in addressing the array of impairments that are resistant to treatment in the chronic phase. Given current knowledge regarding the role of dyscoordination, balance impairment, and deconditioning, it is reasonable to consider that treatment content and the dose, including the duration/frequency, were important factors in the SOARS in producing the results of clinically and statistically significant improvement in impairment and function.

### 4.6. Participation in the Long-Duration, Intensive Intervention

We encountered a number of barriers in offering a program five-days/week for six months, which we worked through to resolve. First, though participants encountered obstacles to attend some sessions, problems were solved, and the average attendance was 3.3 visits/week. Second, due to severe weakness and deconditioning, participants were able to begin with only small challenges. For example, initially, some were able to cycle only 3 min, and with no resistance, for some weeks, before being able to progress at all. The long-duration protocol afforded the opportunity to build strength and improve endurance considerably. In this manner, these chronic stroke survivors with moderate to severe impairment safely and feasibly participated in SOARS, a long-duration, comprehensive mobility and fitness training program that meets recent guideline recommendations but is not current standard practice [5]. This is important because, without intervention, function and quality of life deteriorate during the years after stroke [6,7,9]. The existing standard therapy model provides treatment, for the most part, only in the acute and subacute phases of stroke, which is problematic because more than 33% of stroke survivors remain disabled in the chronic phase [1,2,6]. Physical activity level in chronic stroke survivors is low and associated with poor physical function, fatigue, falls, poor falls self-efficacy, poor balance self-efficacy, depression, and poor health-related quality of life (HR-QOL) [58]. The results of the current study support the viability and usefulness of a long-term mobility/fitness program in terms of clinically significant gains that were realized in impairment, functional mobility, and personal milestones.

### 4.7. Limitations

Of course, in this preliminary, small convenience sample, generalizability is not the next step; rather, a larger and perhaps randomized controlled trial would serve to identify efficacy and provide some useful generalizability. This current preliminary work provides beginning information upon which to build the direction of inquiry. Insurance coverage for a long-term mobility/fitness program is nonexistent in the current health care milieu, which is driven by short-term profits. It is difficult to obtain research funding that covers testing of a costly, long-duration mobility/fitness program. Therefore, it is difficult to obtain the scientific evidence supporting the long-term efficacy and cost savings of a long-term care program. This was a preliminary study in which there was a study therapist who administered the treatment and conducted the assessments. In order to maintain as much objectivity as possible, the scores from the baseline assessment were not visualized during the 6 month assessment; thus, there was a six-month interval between assessments. Given the array of measures, the memory would be taxed to recall prior scores. Still, the fact that the same individual filled both roles means that there could be bias introduced by this study team configuration, giving reason to interpret the results with some caution. At the same time, subject report of milestones is consistent with the quantitative results.

### 4.8. Clinical Implications

Ultimately, from a clinical standpoint, we are seeking improved life-long care of stroke survivors. The current preliminary work is a first step in obtaining the evidence needed for improved mobility and fitness rehabilitation and life-long maintenance. First, the study shows that stroke survivors with persistent mobility deficits and poor endurance can participate in daily intensive rehabilitation sessions (1–2 h) in the chronic phase after stroke. Second, the study shows that stroke survivors in the chronic phase do have further “rehab potential” that should be realized; that is, even after standard rehabilitation, they can still respond to neurorehabilitation showing gains in impairment, function, and quality of life. Third, given that each participant showed a different pattern of recovery according to impairment and function measures, the results for this cohort provide beginning evidence to support the administration of “precision medicine” in the realm of neurorehabilitation (i.e., customized to the individual). Fourth, this “precision” neurorehabilitation, should include the possibility that the therapist can select from as broad a treatment array as needed in order to address the breadth and particular constellation of impairments and dysfunction present in a given stroke survivor. This study is a first step, dependent upon future larger studies to provide justification for the improved live-long care of stroke survivors.

## 5. Conclusions

The dose and the content of the intervention proved feasible for a convenience sample of chronic stroke survivors (intervention array aimed to address impairments underlying mobility dysfunction). The protocol produced personal milestone achievement and moderate to large effect sizes of clinically and statistically improved function beyond that reported, to date, for chronic stroke survivors. These results provide some evidence upon which to embark on a larger study which may provide justification for improved clinical care during the chronic phase after stroke.

## Figures and Tables

**Table 1:** Participant Characteristics.

Subject	Age	Gender	Time Since Stroke (months)	Stroke Location/Details
1	59	male	24	L middle cerebral artery (MCA), ischemic
2	54	female	124	L basal ganglia, hemorrhagic
3	65	male	4	R MCA, ischemic
4	80	male	20	L internal capsule, ischemic
5	63	male	21	L basal ganglia; L frontal, parietal, and occipital cortices; R inferior frontal cortex, ischemic
6	53	female	8	L lateral thalamus, posterior limb of L internal capsule, ischemic
7	60	female	71	L MCA, ischemic
8	46	female	7	R parietal lobe and ventricle, hemorrhage

**Table 2:** Statistically Significant Improvement in Balance (BBS), Joint Movement Coordination (FM), Gait Speed, Functional Mobility (TUG), and Functional Activities (FIM).

Measure	Baseline Mean (std) [Range]	Post Treatment Mean (std) [Range]	Change Score (std)	*p*-Value	95% CI	Effect Size
BBS (max = 56)	42.38	50.25	7.88 (6.05)	0.016	(3.62, 12.0)	1.04
	(8.72)	(4.87)				
	[30–51]	[46–56]				
Fugl-Meyer (max = 34)	19.88	23.75	3.88 (3.66)	0.047	(1.12, 6.12)	0.986
	(3.82)	(3.53)				
	[15–31]					
Gait speed (m/s)	0.464	0.669	0.204 (0.207)	0.023	(0.070, 0.353)	0.572
	(0.246)	(0.403)				
	[0.21–1.0]	[0.24–1.5]				
6MWT (ft)	651	772	121 (148)	0.062	(24.5, 231)	0.238
	(489)	(463)				
	[249–1881]	[269–1800]				
TUG (s)	26.73	17.88	−8.85 (4.28)	0.008	(−12.0, −6.11)	0.772
	(12.07)	(9.18)				
	[12.3–51.5]	[5.8–23.1]				
FIM (max = 126)	112.88	118.88	6.00 (6.54)	0.008	(2.38, 11.1)	0.831
	(7.47)	(5.95)				
	[102–125]	[109–126]				
CHART (max = 600)	465.84	515.83	50.0 (57.85)	0.078	(11.6, 92.0)	0.655
	(80.65)	(60.78)				
	[385.7–600]	[432.7–600]				

Key: BBS: Berg Balance Scale; FM: Fugl-Meyer Limb Coordination Scale; 6MWT: Six Minute Walk Test; TUG: Timed Up and Go; FIM: Functional Independence Measure; CHART: Craig Handicap Assessment and Rating Tool; Std: standard deviation, Ft: feet; S: seconds; CI: confidence interval.

**Table 3:** Clinical and Measurement Benchmarks for Study Measures.

A. Measure	B. Mean Change Score (std)	C. MCID (Clinical Bench-Mark)	D. Percent (%) Who Exceeded MCID	E. MDC (Measurement Bench-Mark)	F. Percent (%) Who Exceeded MDC	G. Functional Threshold	H. Percent (%) Who Exceeded Threshold
BBS (max = 56)	7.9 (6.1)	-	-	4.13	75%	45 (functional independence)	75%, of those with < 45 points at baseline
Fugl-Meyer (max = 34)	3.9 (3.7)	-	-	3.57	75%	-	-
Gait speed (m/s)	0.20 (0.21)	0.16	50%	0.18	38%	Transitioned to higher amb category	25
6MWT (ft)	121.0 (148)	112.86	50%	120	50%	-	-
TUG (s)	−8.9 (4.3)	-	-	3.16	88%	14 (fall risk)	38
FIM (max = 126)	6.0 (6.5)	22	13%	-	-	-	-
CHART (max = 600)	50.0 (57.85)	-	-	-	-	425.83 (norm for chronic stroke)	100

Key: BBS: Berg Balance Scale; FM: Fugl-Meyer Limb Coordination Scale; 6MWT: Six Minute Walk Test; TUG: Timed Up and Go; FIM: Functional Independence Measure; CHART: Craig Handicap Assessment and Rating Tool; m/s: meters/second; MCID: minimal clinically important difference (change); MDC: minimum detectible change.

**Table 4:** Sessions Attended (120 possible, 6 months, 5 sessions/week).

Subject Number	Total Sessions Attended	Mean Per Month	Mean PER Week	Issues Encountered and Dealt with in Order to Attend	Percent of Total 120 Available
6	97	16.20	4.00	Transportation	81%
5	96	16.00	4.00	Depression	80%
2	84	14.00	3.50	Working	70%
1	78	13.00	3.25	Nutrition issues, Transportation (bus)	65%
3	77	12.80	3.20	Working	64%
7	77	12.80	3.20	Cancer Diagnosis and breast Radiation Treatment	64%
4	62	10.33	2.58	Difficulty with ADL’s	52%
8	61	10.12	2.54	Unsupportive Spouse, Transportation	51%
Group Mean (std)	79 (+/−12)	13.2 (+/−2.1)	3.3 (+/−0.52)		66% (+/−10.4)

Key: ADL: activities of daily living; std: standard deviation.

## Supplementary Materials

The following are available online at https://www.mdpi.com/2076-3425/10/8/555/s1. Figure S1: Schema showing hierarchy of motor task difficulty for motor tasks used in Gait Coordination Protocol to restore gait coordination. Schema was used in assessment and treatment progression. Figure S2: Gait Coordination Protocol: framework and tools. BWS = body-weight support, BWSTT = body-weight supported treadmill training, FES = functional electrical stimulation, TT = treadmill training. Figure S3: (a) Initial position for practicing volitional activation of knee flexor muscles. This is within-synergy position for knee flexion practice; that is, hip and ankle are flexed. (b) Volitional activation of knee flexors can result in abnormal simultaneous activation of hip and ankle joint flexors, producing mass limb flexion pattern. If this is the only condition under which practice of volitional knee flexor muscle activation can occur, then this practice paradigm was the initial motor task for knee flexor muscle retraining. Figure S4: (a) Out-of-synergy start position for hip and ankle during coordination task of knee flexion practice out of synergy. This is considered out-of-synergy exercise position because task is knee flexion, whereas contiguous joints are expected to remain in extension (ankle) or neutral (hip) positions throughout knee flexion task. (b) Out-of-synergy knee flexion task demands knee flexion movement, while maintaining hip and ankle in static start positions of extension or neutral, shown respectively at ankle and hip in (a). In (b), participant has succeeded in volitional knee flexion while maintaining ankle in plantar flexion, but was not able to maintain hip in neutral start position; instead, hip flexed unintentionally and abnormally during knee task. Figure S5: (a) Initial position for motor task of alternating knee flexion/extension. Task is to perform full range hip, knee, and ankle flexion followed by full extension. (b) Success in full knee and ankle extension, but only partial success in hip movement, failing to move beyond neutral position into full hip extension, Initial- to midstance phase knee control practice of left involved limb. Figure S6: (a) Participant in midst of initial weight acceptance on left involved limb. (b) End of motor task, at which time body weight has been fully accepted on involved limb, attempting to maintain center of mass over stance foot. Attention was directed to practice of knee joint control during this subphase of stance phase. Figure S7: Training alternating, reciprocal knee flexion/extension movement of right and left limbs in prone position. (a) Task is to maximally flex one limb while other extends, and then (b) reverse movements. Foci of task practice: no compensatory movements, smooth movements, synchronization of timing of right and left movements so that maximal reciprocal flexion and extension movements are reached simultaneously for both limbs.
