# Supplementary material for: Innovative Long-Dose Neurorehabilitation for Balance and Mobility in Chronic Stroke: A Preliminary Case Series
Source: Brain Sci. 2020 Aug 14;10(8):555. doi: 10.3390/brainsci10080555 (PMC7464211; doi:10.3390/brainsci10080555)
Supplement: Supplementary file 1 [file brainsci-10-00555-s001.pdf]

JRRD Guest Editorial

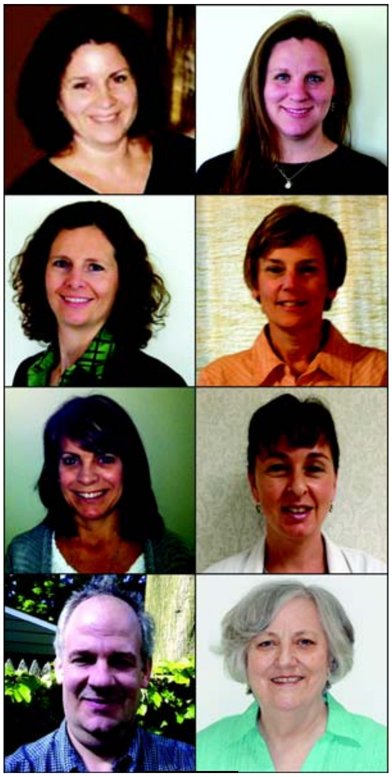

Janis J. Daly, PhD, MS; Jessica P. McCabe, MPT; Jennifer Gansen, DPT; Jean Rogers, PT; Kristi Butler, MPT; Irene Brenner, MSPT; Richard Burdsall, PT; Joan Nethery, MA, PT, NCS

Gait coordination protocol for recovery of coordinated gait, function, and quality of life following stroke

INTRODUCTION

The Gait Coordination Protocol (GCP) was successful in producing clinically and statistically significant gains in impairment, function, and life-role participation for those in the chronic phase after stroke who had exhibited persistent moderate to severe gait deficits [1–3]. The GCP was initially developed to test response to functional electrical stimulation (FES); notably, the GCP produced enhanced coordinated gait regardless of whether FES was used [1]. (The **Video** shows gait recovery in response to the GCP for a participant from the "No-FES group.") In response to national and state presentations, there was a strong call for description of the details of the GCP and its clinical implementation. Therefore, the purpose here is to detail the implementation of this treatment protocol.

We constructed the GCP based on the phenomenon of brain plasticity and associated motor learning principles that included task-specific practice (practice as close-to-normal movement as possible [4–7] with continual progression toward normal), high repetition of the desired movement pattern [8–10], focused attention on the part of the learner [11], training specificity [9,12–14], and awareness and feedback [15]. The GCP, described subsequently, was provided 1.5 h/d, 4 d/wk, and for at least 48 visits (12 weeks) [1–2].

Before treatment, the initial assessment included muscle strength, coordination, muscle tone, balance, gait coordination, gait speed, function, and quality of life. Critical aspects of the assessment included the ability to generate normal movement at the hip, knee, and ankle for the motor tasks listed in **Figure 1**: A–J. For those movements that were impaired, assessment included not only the ability to volitionally generate each of these movements, but also the ability to move in conjunction with an array of assistive movement devices (body-weight support [BWS], body-weight supported treadmill training [BWSTT], FES) and gait support devices (parallel bars, walker, cane).

Each motor task (**Figure 1**) was assessed for the following characteristics:

- Percentage of the normal range of movement that could be executed, volitionally and independently.
- Percentage of the motor task that could be executed with the support of verbal or tactile facilitation.
- Percentage of the normal range of movement that could be executed along with an assistive movement device.
- Normality of effort level during the task (e.g., holding breath, abnormal co-contraction of muscles distant from the targeted task joints or antagonist muscle contractions).
- Compensatory strategies employed during execution of the motor task.
- Percentage of the task for which compensatory strategies were employed.

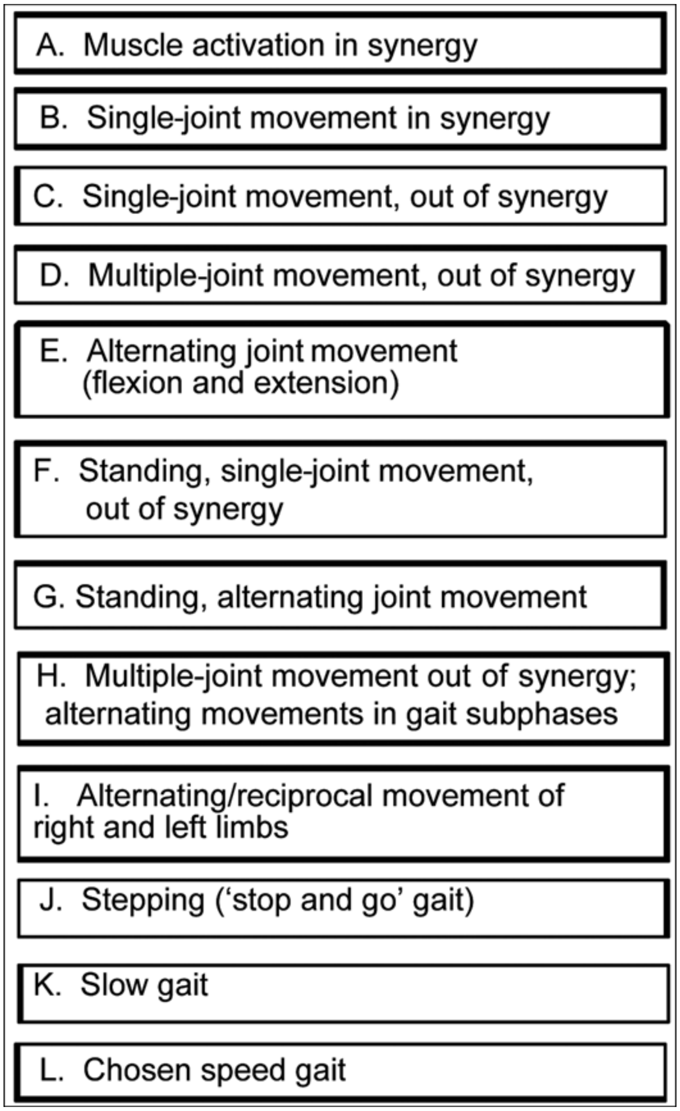

Figure 1.
Schema showing hierarchy of motor task difficulty for motor tasks used in Gait Coordination Protocol to restore gait coordination. Schema was used in assessment and treatment progression.

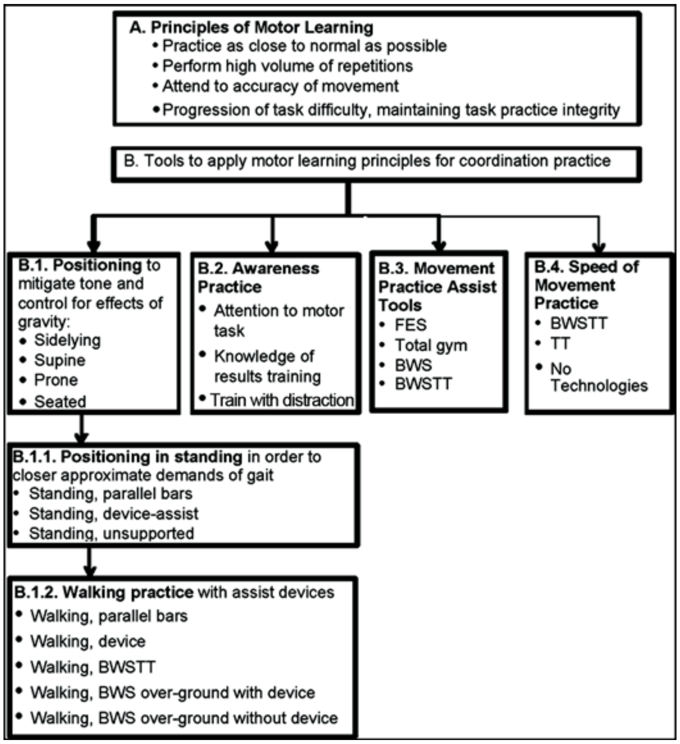

Figure 2.
Gait Coordination Protocol: framework and tools. BWS = body-weight support, BWSTT = body-weight supported treadmill training, FES = functional electrical stimulation, TT = treadmill training.

- Number of repetitions of the motor task that could be performed with only a "beat" between repetitions before the motor task was performed in an abnormal fashion.

In this manner, the assessment was used to identify the initial training level in the hierarchy schema of difficulty (**Figure 1**). For those motor tasks that could not be volitionally performed, an array of motor learning tools were employed (**Figure 2**).

The GCP utilized an array of tools to optimally apply the principles of motor learning. These tools included selective body positioning (**Figure 2**: B.1–B.1.1), awareness training (**Figure 2**: B.2), and practice-assist devices (**Figure 2**: B.3). The position for motor task practice was selected to satisfy practice of both the most normal movement possible and also the most challenging body position, both of which are required for motor skill acquisition [16–19] (**Figure 2**: B.1). Practice included the standing position (**Figure 2**: B.1.1) and dynamic walking, of course, to satisfy the learning principle of task specificity (**Figure 2**: B.1.2).

Awareness training (**Figure 2**: B.2) consisted of learning to identify and differentiate between normal coordinated movement and the abnormal movement, learning to monitor oneself during practice, assessing how close one's movement was to the normal movement pattern, and assessing the frequency of execution of the more normal movement pattern during a series of practice trials [15].

When volitional movement is abnormal, repeating it during practice is counterproductive. In that case, movement-assist, practice devices were used to provide practice of a more normal movement (**Figure 2**: B.3) [16,20–23].

These practice-assist devices included BWS, BWSTT, and FES. These practice-assist technologies allowed study participants to practice coordinated movements both discretely [24] and continuously [24–25] and sooner in the rehabilitation process than otherwise possible with volitional effort alone [16,20].

Finally, speed of movement practice was progressed. As capability for performing a given movement pattern improved in terms of intended muscle activation and sequencing of muscle activations, the practice speed of the movement pattern was increased. The practice of increased speed of movement was incorporated at all levels of coordination difficulty. Tools such as FES and BWSTT were utilized to enhance faster speed practice.

Treatment was progressed in the GCP according to the progression rules shown in the **Table** and the difficulty hierarchy schema for the motor tasks in **Figure 1**. The following sections provide details for implementing the GCP. For clarity, we provide an example as a vehicle to greater understanding. The example here is retraining for impaired knee control coordination during the swing and stance phases of gait for an individual with severe impairment.

IMPLEMENTATION

Training Volitional Muscle Activation/Deactivation Within Synergy (Figure 1: A)

For one who is unable to activate the knee flexor or extensor muscles in any position (manual muscle testing grade of zero in any position), the first treatment goal is to facilitate and elicit volitional muscle activation. In our prior work [16,19], we found that for severe paresis, the side-lying, within-synergy position of the lower limb produced the greatest likelihood of eliciting volitional muscle activation; for knee flexion muscle-activation training, this body position entails side-lying with the entire limb in a within-synergy position (i.e., a flexor synergy position with the hip and ankle flexed). The involved limb was uppermost and supported on an exercise board in the horizontal plane (**Figure 3(a)**).

In this position, abnormal muscle tone was mitigated [16,19] and volitional muscle activation could be more likely achieved. The initial motor task was volitional activation of knee flexors, and initially, flexion at the hip and ankle would be “allowed” until the participant was able to progress to isolated muscle activation exclusively of the knee flexors (**Figure 3(b)**). For some participants with severe impairment, volitional knee flexor muscle activation could be performed under this condition, whereas in other body and/or limb positions, it was not possible [16,19]. In order to retrain this capability, the practice-assist tools were employed (**Figure 2**).

The same principles described for flexion were also used for training volitional knee extensor muscle activation. But the difficulties regarding control of knee extensors are often different; that is, after stroke, the quadriceps muscle group can exhibit unintentional overactivity. In that case, the training included learning to volitionally deactivate the quadriceps muscle group. If volitional deactivation is impaired, it is necessary to train deactivation of the quadriceps muscle early in treatment to restore normal control of agonist/antagonist muscles for the knee joint flexion and extension movements required in the normal gait pattern.

Training Single-Joint Movement Within Synergy (Figure 1: B)

After some recovery of volitional muscle activation/deactivation, the task was progressed to isolated movement at a single joint. An example is volitional knee flexion while maintaining hip and ankle in a static position, within synergy (i.e., hip and ankle in a flexed position; **Figure 3**: A). In contrast to knee flexion practice, knee extension movement was practiced with the hip and ankle in an extended static position to satisfy the within-synergy parameter for knee extension practice.

The clinician provided only the minimal amount of assistance needed, continually reducing external manual or device assistance as the participant regained volitional control. Also, training of knee flexion within synergy was progressed to other body positions, such as seated in a chair with the foot on the floor, the hip and ankle

Table.
Guidelines for motor task progression.

Step	Guideline
1	50 percent of normal range of movement is executed, volitionally, independently; *or* 50 percent of motor task is executed with support of verbal or tactile facilitation; *or* 50 percent of normal range of movement is executed, along with motor assist device.
2	Normal level of effort is expended during task (no holding breath or associative reactions in other limbs or trunk; relaxed uninvolved muscles).
3	If motor compensatory strategies are employed, less than 10 degrees of movement is performed that is compensatory in nature.
4	If motor compensatory strategies are employed, at least half of motor task is performed without compensatory strategies.
5	Five or more repetitions of motor task can be performed in a row with only a “beat” between before motor task deteriorates into uncoordinated or incorrect fashion.

(a) (b)

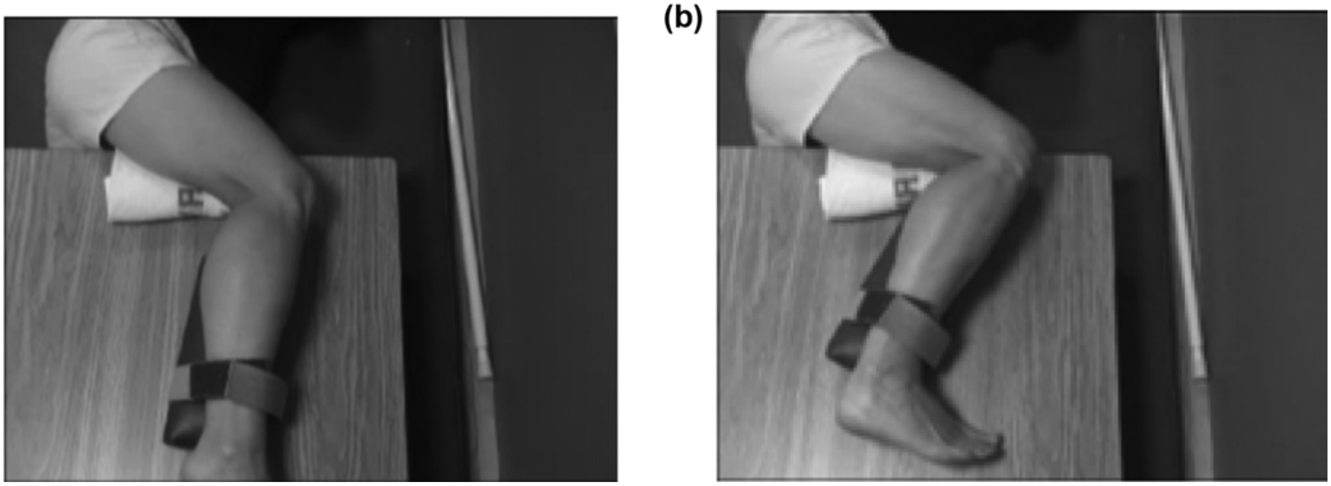

Figure 3.
(a) Initial position for practicing volitional activation of knee flexor muscles. This is within-synergy position for knee flexion practice; that is, hip and ankle are flexed. **(b)** Volitional activation of knee flexors can result in abnormal simultaneous activation of hip and ankle joint flexors, producing mass limb flexion pattern. If this is the only condition under which practice of volitional knee flexor muscle activation can occur, then this practice paradigm was the initial motor task for knee flexor muscle retraining.

flexed, and the knee positioned in 30°–45° of flexion. In this example, the task was to flex the knee beyond 90° by sliding the foot under the chair.

Training Isolated Single-Joint Movement Out of Synergy (Figure 1: C)

With recovery of consistent activation of the paretic muscle(s) within synergy, training was progressed to lesser within-synergy positions. For example, in the knee flexion task, the initial position (whole limb flexed) was progressed to the following: hip in neutral position (0° flexion) and the ankle in neutral or plantar flexion (i.e., extension; **Figure 4(a)**). The motor task in this case was to achieve volitional, isolated, single-joint knee flexion movement with the nonmoving limb joints statically positioned and maintained in an extended position, i.e., out of synergy; **Figure 4(b)** shows success during knee flexion for the coordination task of maintaining the ankle in plantar flexion, but only partial success at maintaining the hip in neutral or extension (the hip unintentionally abnormally moved into partial flexion).

By the same token, the task "out-of-synergy knee extension" was practiced with the hip and ankle positioned and maintained in full flexion.

Knee flexion difficulty was progressed to a prone position with the hip in neutral (**Figure 2**: B.1). The coordination task goal was "knee flexion to 90° while maintaining the hip in neutral (no hip flexion)." Initially, a participant may not be capable of concentric knee flexor muscle contraction in this position, even within a limited range of motion. In that case, training began with an isometric muscle contraction in starting positions of varying degrees of knee flexion. To progress the motor practice, an eccentric muscle contraction of the knee flexors was performed by the participant volitionally lowering the shank to the mat. Finally, practice was progressed to a concentric knee flexor muscle contraction through a progressively greater range of motion until isolated knee flexion was performed through 90° in the prone position.

Training Multiple-Joint Movement Out of Synergy (Figure 1: D)

After some mastery of out-of-synergy, single-joint movement, practice was progressed to out-of-synergy, multiple-joint movement. For the knee flexion training example, the motor task was as follows: simultaneously flex the knee and extend the hip and/or ankle. This motor task was initially practiced in the side-lying position with the involved limb uppermost and supported on an exercise board in the horizontal plane (**Figure 2**: B.1). Training was progressed to more challenging body and limb positions, as described previously and listed in **Figure 2**: B.1.

Training Alternating-Joint Movement (Figure 1: E)

In parallel with the training described for the **Figure 1**: D coordination task, training was conducted for control of alternating knee flexion and knee extension (**Figure 1**: E). Participants had difficulty activating an agonist

(a) (b)

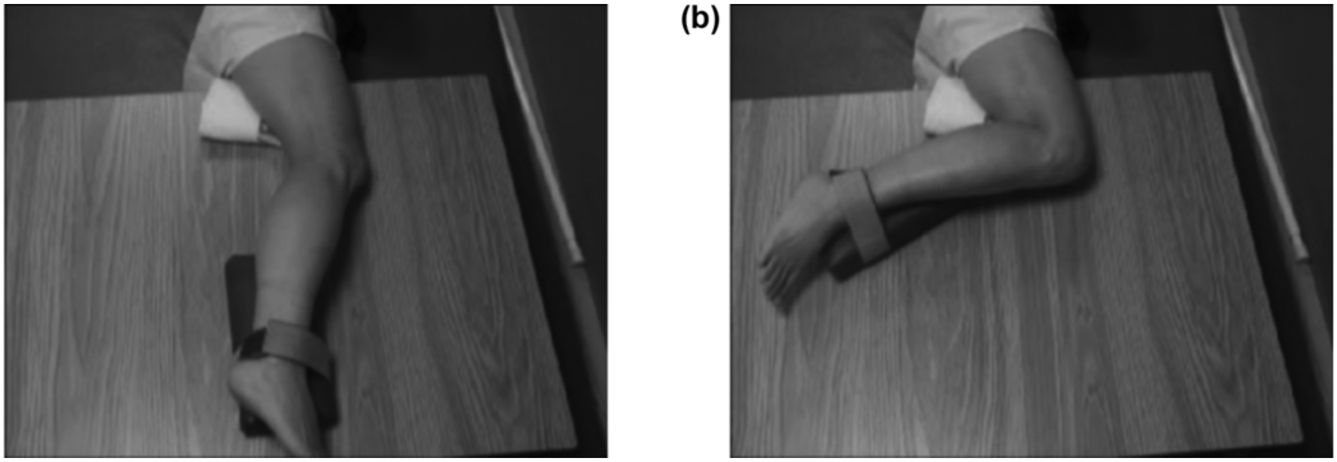

Figure 4.
(a) Out-of-synergy start position for hip and ankle during coordination task of knee flexion practice out of synergy. This is considered out-of-synergy exercise position because task is knee flexion, whereas contiguous joints are expected to remain in extension (ankle) or neutral (hip) positions throughout knee flexion task. **(b)** Out-of-synergy knee flexion task demands knee flexion movement, while maintaining hip and ankle in static start positions of extension or neutral, shown respectively at ankle and hip in **(a)**. In **(b)**, participant has succeeded in volitional knee flexion while maintaining ankle in plantar flexion, but was not able to maintain hip in neutral start position; instead, hip flexed unintentionally and abnormally during knee task.

muscle group, while concomitantly relaxing the antagonist muscle group. For this task, the initial practice position for knee joint movements for the most severely impaired was side-lying with the hip in neutral. This coordination task was to alternately flex and extend the knee through full range while also flexing and extending the hip and knee (**Figure 5**).

Three progressively more challenging training positions included supine on a mat table with the affected limb positioned in 90° of knee flexion with the foot on the floor (Thomas Test Position), prone on a mat, and seated with the thigh supported. As performance improved, the challenge was increased by increasing the speed of volitional, alternating knee joint movements. To further progress the difficulty level of exercises, out-of-synergy hip and ankle movements were added (e.g., hip extension/knee flexion/ankle plantar flexion followed by hip flexion/knee extension/ankle dorsiflexion).

(a) (b)

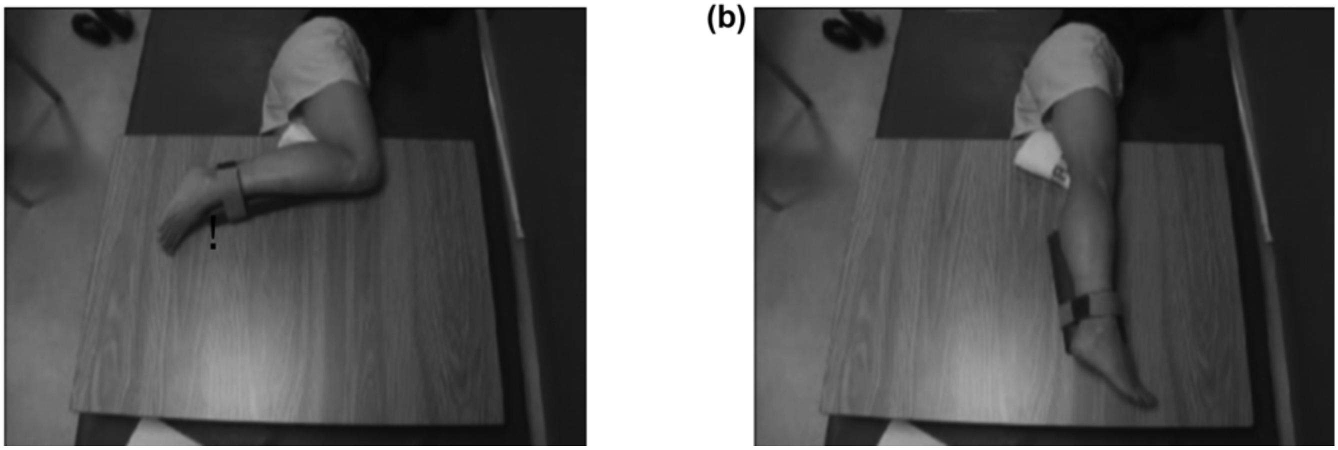

Figure 5.
(a) Initial position for motor task of alternating knee flexion/extension. Task is to perform full range hip, knee, and ankle flexion followed by full extension. **(b)** Success in full knee and ankle extension, but only partial success in hip movement, failing to move beyond neutral position into full hip extension.

Training Standing Single-Joint Movement Out of Synergy (Figure 1: F)

Knee joint movement was practiced as a single-joint movement in the standing position. The initial position for practicing the standing knee flexion task was as follows: all body weight borne on the uninvolved limb (using parallel bars) and the involved limb flexed at the knee with the toe resting on the floor behind the body with the hip in neutral (out of synergy for a knee flexion task). The task was to flex the knee, maintaining the hip in neutral (no hip flexion). Training was progressed in three ways: (1) increasing the amount of hip extension in the initial position, (2) using less support (e.g., minimizing use of parallel bars or using a cane), and (3) increasing the range of knee flexion movement.

Training Standing, Weight-Bearing, Alternating-Joint Movement (Figure 1: G)

For stance-phase knee control, the motor task was progressed to alternating knee flexion and extension joint movements. Task practice included progression to small-range knee flexion/extension movements at the end of the knee extension range (shallow knee bends); this task was progressed from bilateral to unilateral weight bearing and by incrementally decreasing upper-limb support. The task demand was to achieve graded, smooth control of knee flexion and extension movements during full-body weight bearing and without abnormal knee hyperextension or hyperflexion.

Training Multiple-Joint Movement Out of Synergy in Gait Subphases of Stance (Figure 1: H)

In the stance phase, knee control is required in the subphases of loading response (**Figure 6(a)**), midstance (**Figure 6(b)**), and late stance. In each subphase, there are unique demands of knee movement and knee position control. Therefore, each subphase of stance was practiced separately. During training, particular attention was focused on the training of sequencing and grading of muscle contractions across the given subphase.

Training Alternating, Reciprocal Movement of Right and Left Limbs (in Positions of Prone, Sitting, Stepping) (Figure 1: I)

The gait pattern requires independent movement of the right and left limbs. The practice positions for alternating reciprocal movement included supine, prone (**Figure 7**), sitting, and stepping in place.

Alternating limb-movement practice was progressed in a number of ways, including the following: increasing the range demands (i.e., flexing and extending through greater movement excursions), increasing the number of joints to be controlled (e.g., for knee flexion practice, adding hip and ankle movements), more challenging positions (sitting, standing), increasing the number of repetitions, and increasing the speed of movement practice.

Training Stepping ("Stop-and-Go" Gait) (Figure 1: J)

A targeted stance-phase gait component can be practiced within the framework of one stride. For the subphase

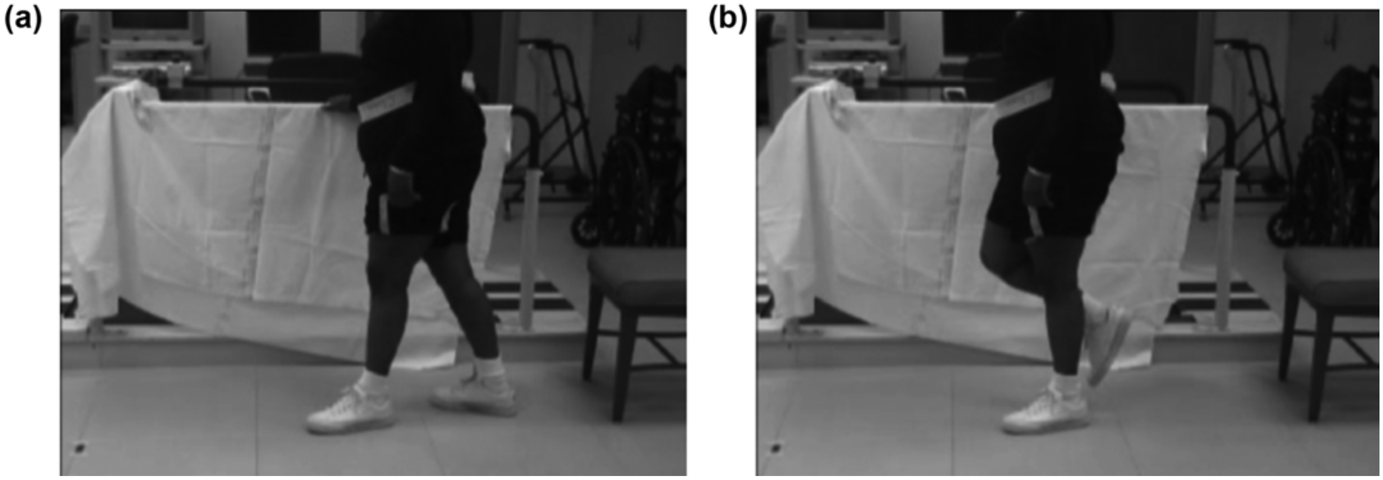

Figure 6.
Initial- to midstance phase knee control practice of left involved limb. **(a)** Participant in midst of initial weight acceptance on left involved limb. **(b)** End of motor task, at which time body weight has been fully accepted on involved limb, attempting to maintain center of mass over stance foot. Attention was directed to practice of knee joint control during this subphase of stance phase.

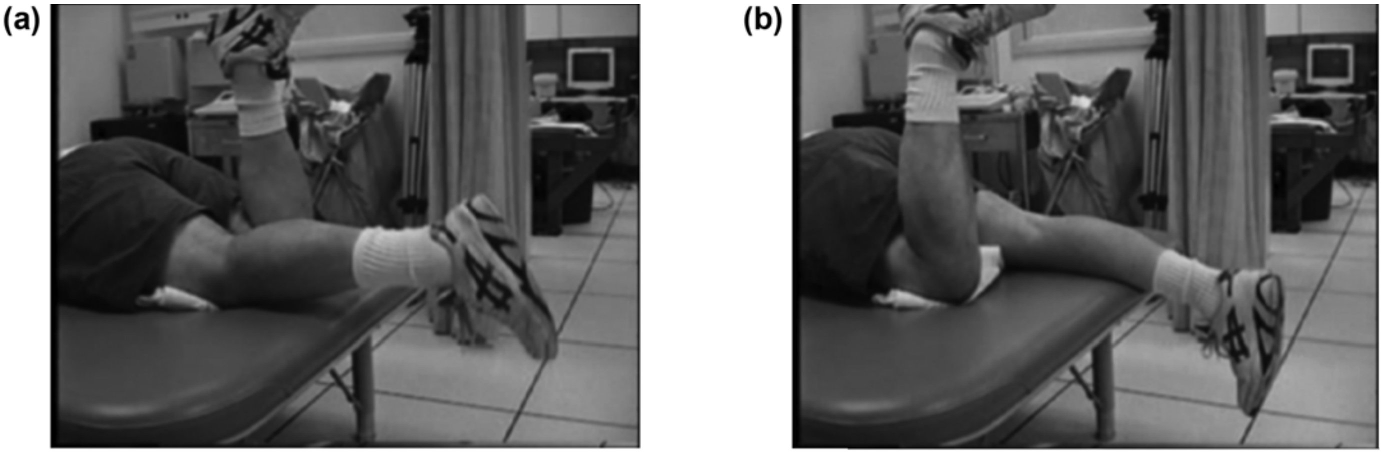

Figure 7.
Training alternating, reciprocal knee flexion/extension movement of right and left limbs in prone position. **(a)** Task is to maximally flex one limb while other extends, and then **(b)** reverse movements. Foci of task practice: no compensatory movements, smooth movements, synchronization of timing of right and left movements so that maximal reciprocal flexion and extension movements are reached simultaneously for both limbs.

of loading response to midstance, the sequence of the practice task was as follows: initial position, in which full weight is borne on the uninvolved limb; shift weight forward to the involved limb (loading response phase, with knee extended) and practice knee flexion to 15°; complete the entire stride; and begin a new stride, with conscious attention again directed to that targeted subphase and knee extension followed by smooth knee flexion of about 15° during weight bearing. During the next subphase, as the center of mass progresses forward, a different challenge is practiced: moving from 15° of knee flexion to full knee extension during single-limb weight bearing. The same stop-and-go procedure can be applied, but this time, the conscious practice is directed to smooth knee extension at and just after midstance phase.

Training Slow Gait (Fluid Gait) (Figure 1: K)

For practice of the targeted gait component within the gait pattern, slow walking was first practiced so that conscious attention was directed to the single targeted gait component each time it occurred during slowly performed, uninterrupted steps across an even surface.

Training Chosen-Speed Gait (Figure 1: L)

The targeted gait component was practiced at the chosen speed that was comfortable. As in slow gait practice, conscious attention was directed to the single targeted gait component each time it occurred during uninterrupted steps across an even surface.

We incorporated movement-assist devices and technologies into the GCP. **Figure 1**: B lists a number of technologies that were used in the GCP. FES can be used in all aspects of training including exercise, BWSTT, and overground gait training. Technologies were incorporated into the GCP to provide the participant with an opportunity to practice a given motor task as close to normal as possible for as many repetitions as possible when volitional effort did not produce such practice opportunities. Importantly, the assistance of technologies was progressively reduced as quickly as the participant regained volitional control of coordination sufficient to practice a close-to-normal coordination task.

CONCLUSIONS

We can note that the current GCP offers clinical application of the motor learning principles associated with brain plasticity that have been separately established as evidence based. Additionally, we formulated a motor task schema of increasingly difficult motor practice. Within that framework and those principles, the GCP integrates the use of strategies and tools into a cohesive protocol that can engage the motor learning principles to such an extent that coordination can be progressively improved to satisfy the goal of improving gait coordination. This protocol is the first gait coordination training method, to our knowledge, to synthesize and integrate gait training in such a manner and to be quantitatively tested [1]. In response to this GCP, the significant improvements in impairments and coordinated gait exhibited a high effect size and so were sufficiently robust to produce a

statistically significant improvement in measures of functional activities and meaningful life role participation activities [2]. These results together suggest that the judicious application of motor learning principles can produce clinically important gait coordination recovery for those with otherwise persistent gait deficits after stroke.

Janis J. Daly, PhD, MS;[1*] Jessica P. McCabe, MPT;[2] Jennifer Gansen, DPT;[2] Jean Rogers, PT;[2] Kristi Butler, MPT;[2] Irene Brenner, MSPT;[2] Richard Burdsall, PT;[2] Joan Nethery, MA, PT, NCS[2]

[1]Brain Rehabilitation Research Center of Excellence, Malcom Randall Gainesville Department of Veterans Affairs Medical Center (VAMC), Gainesville, FL; Department of Neurology, College of Medicine; University of Florida, Gainesville, FL; and Brain Rehabilitation Research Program, McKnight Brain Institute, University of Florida, Gainesville, FL; [2]Cognitive and Motor Learning Research Program, Louis Stokes Cleveland VAMC, Cleveland, OH

[*]Email: jjd17@case.edu

ACKNOWLEDGMENT

Funding/Support: This material was based on work supported by the Department of Veterans Affairs, Office of Rehabilitation Research and Development (grants B5080S, A3102R, and B2226R).

REFERENCES

1. Daly JJ, Zimbelman J, Roenigk KL, McCabe JP, Rogers JM, Butler K, Burdsall R, Holcomb JP, Marsolais EB, Ruff RL. Recovery of coordinated gait: randomized controlled stroke trial of functional electrical stimulation (FES) versus no FES, with weight-supported treadmill and over-ground training. Neurorehabil Neural Repair. 2011;25(7):588–96. [PMID:21515871] http://dx.doi.org/10.1177/1545968311400092
2. Pundik S, Holcomb J, McCabe J, Daly JJ. Enhanced life-role participation in response to comprehensive gait training in chronic stroke survivors. Disabil Rehabil. 2012;34(18):1535–39. [PMID:22324654] http://dx.doi.org/10.3109/09638288.2011.650308
3. Daly JJ, Sng K, Roenigk K, Fredrickson E, Dohring M. Intra-limb coordination deficit in stroke survivors and response to treatment. Gait Posture. 2007;25(3):412–18. [PMID:16824762] http://dx.doi.org/10.1016/j.gaitpost.2006.05.007
4. Biernaskie J, Corbett D. Enriched rehabilitative training promotes improved forelimb motor function and enhanced dendritic growth after focal ischemic injury. J Neurosci. 2001;21(14):5272–80. [PMID:11438602]
5. Jones TA, Chu CJ, Grande LA, Gregory AD. Motor skills training enhances lesion-induced structural plasticity in the motor cortex of adult rats. J Neurosci. 1999;19(22):10153–63. [PMID:10559423]
6. Nudo RJ, Milliken GW, Jenkins WM, Merzenich MM. Use-dependent alterations of movement representations in primary motor cortex of adult squirrel monkeys. J Neurosci. 1996;16(2):785–807. [PMID:8551360]
7. Nudo RJ, Wise BM, SiFuentes F, Milliken GW. Neural substrates for the effects of rehabilitative training on motor recovery after ischemic infarct. Science. 1996;272(5269):1791–94. [PMID:8650578] http://dx.doi.org/10.1126/science.272.5269.1791
8. Bütefisch C, Hummelsheim H, Denzler P, Mauritz KH. Repetitive training of isolated movements improves the outcome of motor rehabilitation of the centrally paretic hand. J Neurol Sci. 1995;130(1):59–68. [PMID:7650532] http://dx.doi.org/10.1016/0022-510X(95)00003-K
9. Dean CM, Shepherd RB. Task-related training improves performance of seated reaching tasks after stroke. A randomized controlled trial. Stroke. 1997;28(4):722–28. [PMID:9099186] http://dx.doi.org/10.1161/01.STR.28.4.722
10. Kimberley TJ, Samargia S, Moore LG, Shakya JK, Lang CE. Comparison of amounts and types of practice during rehabilitation for traumatic brain injury and stroke. J Rehabil Res Dev. 2010;47(9):851–62. [PMID:21174250] http://dx.doi.org/10.1682/JRRD.2010.02.0019
11. Singer R, Lidor R, Cauraugh JH. To be aware or not aware? What to think about while learning and performing a motor skill. Sport Psychol. 1993;7:19–30.
12. Pascual-Leone A, Torres F. Plasticity of the sensorimotor cortex representation of the reading finger in Braille readers. Brain. 1993;116(Pt 1):39–52. [PMID:8453464] http://dx.doi.org/10.1093/brain/116.1.39
13. Elbert T, Pantev C, Wienbruch C, Rockstroh B, Taub E. Increased cortical representation of the fingers of the left hand in string players. Science. 1995;

270(5234):305–7. [PMID:7569982] http://dx.doi.org/10.1126/science.270.5234.305
14. Plautz EJ, Milliken GW, Nudo RJ. Effects of repetitive motor training on movement representations in adult squirrel monkeys: role of use versus learning. Neurobiol Learn Mem. 2000;74(1):27–55. [PMID:10873519] http://dx.doi.org/10.1006/nlme.1999.3934
15. Winstein CJ. Knowledge of results and motor learning—implications for physical therapy. Phys Ther. 1991;71(2):140–49. [PMID:1989009]
16. Daly JJ, Barnickle K, Kobetic R, Marsolais EB. Electrically induced gait changes post stroke, using an FNS system with intramuscular electrodes and multiple channels. J Neuro Rehab. 1993;7(1):17–25.
17. Ryerson S, Levit K. Functional movement reeducation: stroke rehabilitation. New York (NY): Churchill Livingstone, Inc; 1997.
18. Carr J, Shepherd R. Stroke rehabilitation: guidelines for exercise and training to optimize motor skill. Philadelphia (PA): Butterworth-Heinemann, Elsevier Science Limited; 2003.
19. Daly JJ, Roenigk K, Cheng R, Ruff RL. Abnormal leg muscle latencies and relationship to dyscoordination and walking disability after stroke. Rehabil Res Pract. 2011;2011:1–8. [PMID:22110973] http://dx.doi.org/10.1155/2011/313980
20. Daly JJ, Ruff RL, Osman S, Hull JJ. Response of prolonged flaccid paralysis to FNS rehabilitation techniques. Disabil Rehabil. 2000;22(12):565–73. [PMID:11005746] http://dx.doi.org/10.1080/096382800416814
21. Daly JJ, Ruff RL. Electrically induced recovery of gait components for older patients with chronic stroke. Am J Phys Med Rehabil. 2000;79(4):349–60. [PMID:10892621] http://dx.doi.org/10.1097/00002060-200007000-00006
22. Daly JJ, Ruff RL. Feasibility of combining multichannel functional neuromuscular stimulation with weight-supported treadmill training. J Neurol Sci. 2004;225(1–2):105–15. [PMID:15465093] http://dx.doi.org/10.1016/j.jns.2004.07.006
23. Chen G, Patten C. Treadmill training with harness support: selection of parameters for individuals with poststroke hemiparesis. J Rehabil Res Dev. 2006;43(4):485–98. [PMID:17123188] http://dx.doi.org/10.1682/JRRD.2005.04.0063
24. Fugl-Meyer AR, Jääskö L, Leyman I, Olsson S, Steglind S. The post-stroke hemiplegic patient. 1. A method for evaluation of physical performance. II. Incidence, mortality, and vocational return in Göteborg, Sweden with a review of the literature. Scand J Rehabil Med. 1975;7(1):13–31. [PMID:1135616]
25. Lee TD, Genovese ED. Distribution of practice in motor skill acquisition: different effects for discrete and continuous tasks. Res Q Exerc Sport. 1989;60(1): 59–65. [PMID:2489826]

This article and any supplementary material should be cited as follows:
Daly JJ, McCabe JP, Gansen J, Rogers J, Butler K, Brenner I, Burdsall R, Nethery J. Gait coordination protocol for recovery of coordinated gait, function, and quality of life following stroke. J Rehabil Res Dev. 2012;49(8):xix–xxviii.
http://dx.doi.org/10.1682/JRRD.2012.07.0127

ALL SUBMISSIONS SCREENED BY: iThenticate Professional Plagiarism Prevention WANT TO PRE-CHECK YOUR WORK? »
crossref MEMBER CROSSREF.ORG THE CITATION LINKING BACKBONE

This content is in the Public Domain.
